# Natural killer cells from endurance-trained older adults show improved functional and metabolic responses to adrenergic blockade and mTOR inhibition

**DOI:** 10.1038/s41598-025-06057-y

**Published:** 2025-07-14

**Authors:** Luciele Guerra Minuzzi, Helena Batatinha, Christopher Weyh, Vidya Srokshna Balasubramanian Lakshmi, Carmen Fiuza-Luces, Beatriz G. Gálvez, Alejandro Lucia, Ana Maria Teixeira, Natascha Sommer, José Cesar Rosa-Neto, Fabio Santos Lira, Karsten Krüger

**Affiliations:** 1 Exercise and Immunometabolism Research Group, Postgraduation Program in Movement Sciences, Department of Physical Education, São Paulo State University (UNESP), 305 Roberto Simonsen, Presidente Prudente, 19060-900 Brazil; 2 CIPER, Faculty of Sport Sciences and Physical Education, University of Coimbra, Coimbra, Portugal; 3 School of Nutritional Sciences and Wellness, The University of Arizona, Tucson, AZ USA; 4 Department of Pediatrics, The University of Arizona, Tucson, AZ USA; 5 Department of Exercise Physiology and Sports Therapy, Institute of Sport Science, Justus-Liebig University Giessen, Giessen, Germany; 6 Excellence cluster Cardio-Pulmonary Institute (CPI), Justus-Liebig-University Giessen, Giessen, Germany; 7 Physical Exercise and Pediatric Cancer Research Group, Research Institute of the Hospital 12 de Octubre (‘imas12’), Madrid, Spain; 8 Department of Biochemistry and Molecular Biology, Faculty of Pharmacy, Universidad Complutense de Madrid, Madrid, Spain; 9 Department of Sport Sciences, Faculty of Medicine, Health and Sports, Universidad Europea de Madrid, Madrid, Spain; 10 Immunometabolism Research Group, Institute of Biomedical Science, University of São Paulo, São Paulo, Brazil

**Keywords:** Physical exercise, Natural killer, Metabolic reprogramming, Mitochondrial function, Propranolol, Rapamycin, Cellular immunity, NK cells, Biomarkers

## Abstract

Aging is associated with immune dysfunction, but long-term endurance training may confer protective effects on immune cell function. This study investigates how natural killer (NK) cell phenotypes, functional markers, and metabolism differ between endurance-trained and untrained older adults. Ex vivo expanded NK cells from endurance-trained (63.6 ± 2.1 years) and untrained (64.3 ± 3.3 years) males were exposed to adrenergic blockade (propranolol; 0–200 ng/mL) or mTOR inhibition (rapamycin; 10–100 ng/mL), both with or without PMA-induced inflammatory stimulation. Flow cytometry assessed NK subsets, activation (CD38, CD57, CD107a, NKG2D), senescence (KLRG1), and inhibitory markers (PD-1, LAG-3, TIM-3, NKG2A). Seahorse analysis measured metabolic parameters. Trained participants displayed healthier immune profiles (lower NLR, SII) and higher effector NK cells with lower cytotoxic subsets. Propranolol at 100 ng/mL blunted PMA-driven increases in CD57, CD107a, and NKG2D, while potentiating regulatory markers KLRG1, LAG-3, and PD-1 in the trained group, indicating stronger immunoregulation. With rapamycin, trained NK cells preserved NKG2D and CD107a at 10 ng/mL, maintaining cytotoxicity and degranulation. In contrast, at 100 ng/mL rapamycin plus PMA, trained NK cells shifted toward an effector phenotype with higher CD57 and CD107a, yet a blunted PMA-increased LAG-3 and TIM-3, suggesting resistance to exhaustion. PD-1 and KLRG1 remained elevated, reflecting balanced immune control. Mitochondrial analysis revealed that trained NK cells exhibited higher basal and maximal OCR, greater spare respiratory capacity, and OCR/ECAR ratio, reflecting superior metabolic fitness. These findings indicate that endurance-trained older adults have NK cells with greater functional adaptability, reduced senescence, and enhanced metabolism under inflammatory and pharmacological stress.

**Supplementary Information:**

The online version contains supplementary material available at 10.1038/s41598-025-06057-y.

## Introduction

There is robust evidence that long-term endurance training provides better immune defense, resulting in stronger, long-lasting antibody responses to influenza vaccination, improved immune, metabolic, and redox balance, and delayed biological aging^1–5^. Notably, long-term exercise has been shown to delay immunosenescence^3,6–9^ and age-related systemic low-grade inflammation (known as inflammaging)^10,11^essential for sustaining immune function with aging. In turn, the aforementioned effects might be mediated by the influence of exercise on immune cell-released molecules^12–16^.

One key mechanism behind these effects is the exercise-induced release of catecholamines and interleukin (IL)-6, both of which play significant roles in the rapid mobilization of natural killer (NK) cells during and immediately after exercise. NK concentration increases up to fivefold during exercise and returns to baseline levels shortly afterward^17^. Additionally, exercise-induced release of IL-6 and IL-15 activates NK cells and upregulates their metabolic pathways via mTOR complex 1 (mTORC1)-dependent signaling^18^enhancing the release of interferon-gamma (IFN-γ) and granzyme B, thereby increasing their metabolic activity^19,20^. Beta-adrenergic receptors (β-ARs), highly expressed in NK cells, play a central role in these processes, facilitating both metabolic activation and mobilization^21^ through the mTOR pathway^22^. Although these links between exercise-released myokines and immune maintenance are intuitive and have been demonstrated in cross-sectional studies, they have yet to be tested mechanistically^9,23,24^.

In this context, fluctuations in circulating glucose levels during exercise play a central role in adrenergic signaling^25^modulating energetic sensors such as AMPK-activated protein kinase (AMPK), mTOR, and hypoxia-inducible factor 1 alpha (HIF-1α)^26^. However, how NK cell metabolic pathways adapt to energy-limited conditions during exercise remains largely unknown^27^. We hypothesized that, during exercise, NK cells encounter energy-limited conditions, which may favor AMPK activation. Concurrently, mTORC1 activation, stimulated by IL-6 and adrenaline, supports NK cell function in these “economical mode” conditions. Repeated exposure through high-intensity exercise may enhance these adaptations. After exercise, with rest and nutrition, mTOR pathway stimulation furthers NK cell metabolic reprogramming, leading to a more efficient functional profile over time.

The primary objective of this study was to compare NK cell function, metabolic reprogramming, and immune regulation between endurance-trained and untrained older adults. Specifically, we aimed to: (a) assess how β-adrenergic receptor blockade (propranolol) and mTOR pathway inhibition (rapamycin) influence NK cell activation and regulation; (b) compare the immune phenotypes and metabolic responses of NK cells from endurance-trained and untrained older adults; (c) determine the mitochondrial respiration in NK cells under pharmacological and inflammatory challenges in endurance-trained and untrained older adults.

## Results

Compared to untrained individuals, trained participants had lower body weight (*P =* 0.010), BMI (*P =* 0.027), and body fat percentage (*P =* 0.010), while showed higher fat-free mass percentage (*P =* 0.010) and higher peak oxygen uptake (VO_2peak;_ *P <* 0.0001; Table 1). The trained group showed higher lymphocyte percentages (*P =* 0.012), a lower Neutrophil-to-Lymphocyte Ratio (NLR; *P =* 0.030), and a lower Systemic Immune-Inflammation Index (SII; *P =* 0.029) compared to the untrained group (Table 1).

**Table 1:** Comparison of body composition and immunological profiles between untrained and trained older adults.

Variable	Untrained	Trained	t-test
	*N* = 4	*N* = 5	*P*-value
Age (years)	64.3 (3.3)	63.6 (2.1)	0.728
BMI (kg/m^2^)	27.5 (3.6)	22.5 (1.6)	0.027
Fat (%)	27.7 (5.4)	17.9 (2.7)	0.010
Fat-free mass (%)	72.4 (5.5)	82.1 (2.7)	0.010
VO_2max_ (mL/kg/min)	25.0 (3.6)	43.2 (4.0)	0.0001
HOMA-IR index	2.3 (1.0)	1.5 (1.5)	0.399
Cortisol (µg/dL)	14.5 (3.4)	18.1 (4.3)	0.235
Leukocytes (×10^3^/µL)	5.9 (1.1)	5.1 (0.9)	0.280
Platelets (×10^3^/µL)	284.5 (41.0)	237.2 (24.8)	0.068
Lymphocytes (%)	30.4 (3.1)	38.2 (3.7)	0.012
Lymphocytes (×10^3^/µL)	1.8 (0.4)	2.0 (0.5)	0.590
Monocytes (%)	9.9 (2.5)	9.2 (0.6)	0.568
Monocytes (×10^3^/µL)	0.6 (0.2)	0.5 (0.1)	0.336
Segmented neutrophils (%)	54.4 (5.0)	48.7 (5.0)	0.132
Segmented neutrophils (×10^3^/µL)	3.2 (0.5)	2.5 (0.5)	0.079
NLR	1.8 (0.3)	1.3 (0.3)	0.030
PLR	168.1 (62.6)	127.8 (36.3)	0.263
SII	523.3 (157.0)	308.3 (75.2)	0.029

Data are mean (Standard Deviation). Abbreviations: BMI = body mass index; FFM = fat-free mass; HOMA-IR = homeostatic model assessment for insulin resistance; NLR = neutrophil-to-lymphocyte ratio; PLR = platelet-to-lymphocyte ratio; SII = systemic immune-inflammation index. NLR and PLR were defined as the total number of neutrophils (N) or platelets (P) divided by the total number of lymphocytes (L). SII was calculated with the formula SII = (P × N)/L, where P, N, and L refer to peripheral platelet, neutrophil, and lymphocyte counts, respectively.

### The beneficial effects of rhythmically mediated adrenergic stimulation on NK cells in trained individuals

#### Propranolol dose-response effects on NK cell phenotypes, activation, and regulatory markers

**Fig. 1:**
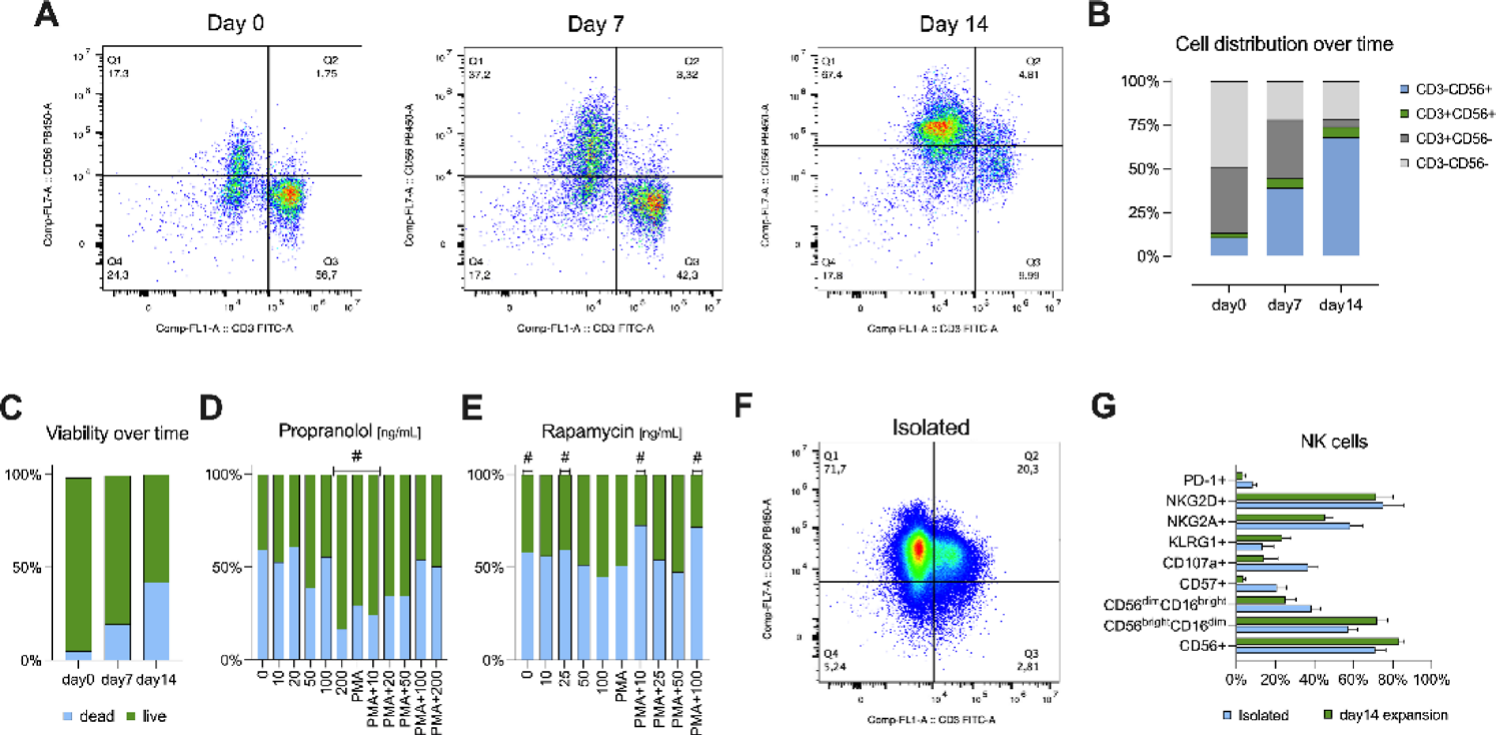
Ex vivo expansion and pharmacological modulation of NK cell viability and phenotype. (**A**) Representative flow cytometer density plots of CD3 versus CD56 expression in cell preparations at days 0, 7, and 14 of the expansion protocol. (**B**) Distribution of viable cells into four CD3/CD56 subsets: CD3⁻CD56^+^ (blue), CD3^+^CD56^+^ (green), CD3^+^CD56⁻ (dark gray), and CD3⁻CD56⁻ (light gray). (**C**) Percentage of viable NK cells at days 0, 7, and 14, determined by live/dead staining (live = green; dead = blue). (**D**,**E**) Dose–response curves for expanded NK cell viability, treated with increasing concentrations of propranolol (D) and rapamycin (E) for 48 h. (**F**) Representative flow cytometry plot of freshly isolated NK cells. (**G**) Comparison of NK cells phenotypes after isolation versus 14 days of expansion. Data are presented as mean ± SEM for *n* = 4–5 independent donors. CD, cluster of differentiation; KLRG1, killer cell lectin-like receptor subfamily G member 1; LAG-3, lymphocyte-activation gene 3; NK, natural killer; NKG2A, natural killer group 2, member A; NKG2D, natural killer group 2, member D; PD-1, programmed death-1.

**Fig. 2:**
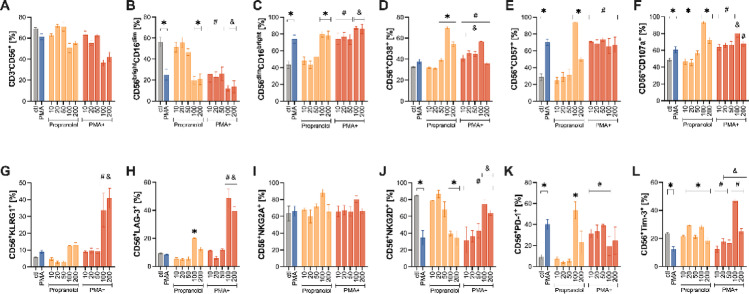
Propranolol dose-response effects on NK cells phenotype, activation, and regulatory receptor expression. Expanded NK cells were treated with increasing concentrations of propranolol (0, 10, 20, 50, 100, or 200 ng/mL) in the absence or presence of an inflammatory stimulus (PMA plus ionomycin, brefeldin A, and monensin). (**A**) Total NK cells (CD3⁻CD56^+^). (**B**,**C**) Cytotoxic (CD56^dim^CD16^bright^) and effector (CD56^bright^CD16^dim^) subset. (**D**–**F**) Activation (CD38), differentiation (CD57), and degranulation (CD107a) markers. (**G**) Senescence marker KLRG1. (**H**–**J**) Regulatory receptors LAG-3, NKG2A, and NKG2D. (**K**–**L**) Immune-exhaustion markers PD-1 and TIM-3. Data are presented as mean ± SEM from *n* = 4 untrained and *n* = 5 endurance-trained donors. * *P* < 0.05 versus untreated control; & *P* < 0.05 versus PMA; # *P* < 0.05 between PMA-stimulated and unstimulated at the same dose. All comparisons were determined using Bonferroni post hoc tests following a significant interaction effect detected by two-way ANOVA. CD, cluster of differentiation; KLRG1, killer cell lectin-like receptor subfamily G member 1; LAG-3, lymphocyte-activation gene 3; NK, natural killer; NKG2A, natural killer group 2, member A; NKG2D, natural killer group 2, member D; PD-1, programmed death-1; PMA, phorbol 12-myristate 13-acetate; TIM-3, T-cell immunoglobulin and mucin-domain containing-3.

Expanded NK cells (Fig. 1A-D) were cultured at 5 × 10^5^ cells/mL with increasing concentrations of propranolol (0-200 ng/mL), either in the absence or presence of the inflammatory cocktail (PMA) to assess propranolol-induced changes in NK cell phenotype (Fig. 2). No significant propranolol × stimulus interaction effects were observed for the frequency of CD3^–^CD56^+^ (*P* = 0.114; Fig. 2A) and CD56^+^NKG2A^+^ (*P* = 0.488; Fig. 2I) cells (Suppl. Table 1).

PMA stimulation alone increased effector phenotypes—including higher frequencies of effector NK cells (*P* = 0.0001; Fig. 2C), CD57^+^ (*P <* 0.0001; Fig. 2E), CD107a^+^ (*P* = 0.016; Fig. 2F), and PD-1^+^ (*P* = 0.003; Fig. 2K)—while reducing cytotoxic NK cells (*P* = 0.0002; Fig. 2B), NKG2D^+^ (*P* = 0.0004; Fig. 2J), and TIM-3+ (*P* = 0.0003; Fig. 2L) NK cells frequencies relative to untreated cells (ctl). When combined with lower propranolol doses (0–50 ng/mL), PMA often further augmented these effector markers beyond either condition alone (Fig. 2).

Nonetheless, higher propranolol doses (100–200 ng/mL) decreased the frequencies of cytotoxic NK cells (*P* < 0.0001; Fig. 2B) and the activating receptor NKG2D^+^ (*P* = 0.0003; *P* = 0.0001; Fig. 2J), while increasing effector NK cells (*P* < 0.0001; Fig. 2C), as well as CD38^+^ (*P* < 0.0001 for both; Fig. 2D), CD57^+^ (*P* < 0.0001, *P* = 0.004; Fig. 2E), and CD107a^+^ (*P* < 0.0001, *P* = 0.0002; Fig. 2F) relative to untreated cells (ctl). Notably, at 100 ng/mL, non-stimulated cultures often exceeded PMA-stimulated frequencies of CD38^+^ (*P =* 0.0002; Fig. 2D), CD57^+^ (*P =* 0.0006; Fig. 2E), CD107a^+^ (*P* = 0.0001; Fig. 2F), and PD-1^+^ (*P =* 0.001; Fig. 2K), indicating that propranolol at these doses may blunt the inflammatory effect of PMA on these markers.

By contrast, KLRG1 and LAG-3 expression were markedly upregulated by PMA in the presence of 100–200 ng/mL propranolol, resulting in higher cell frequencies than propranolol and PMA alone (Fig. 2G-H). Specifically, KLRG1^+^ NK cell frequencies increased with PMA + 100 ng/mL (*P* = 0.007) and PMA + 200 ng/mL (*P* = 0.001) relative to PMA and propranolol at the same doses (*P* = 0.001 for 100 ng/mL; *P* = 0.0001 for 200 ng/mL; Fig. 2G). Similarly, LAG-3^+^ NK cell frequency increased with 100 ng/mL propranolol (*P* = 0.012 vs. control). At 100–200 ng/mL, PMA induced significantly higher LAG-3^+^ NK cell frequencies than propranolol at the same doses (*P* < 0.0001 for both) and PMA (*P* < 0.0001; Fig. 2H).

Conversely, NKG2D + NK cell frequencies were generally elevated under non-stimulated conditions at 0–50 ng/mL (*P* = 0.0004 for PMA, *P* = 0.0007 for PMA + 10 ng/mL, *P* = 0.0004 for PMA + 20 ng/mL, *P* = 0.033 for PMA + 50 ng/mL), but decreased at 100–200 ng/mL relative to control (*P* = 0.0003 and *P* = 0.0001, respectively; Fig. 2J). However, at these higher doses, PMA-stimulated cultures showed greater NKG2D^+^ NK cell frequencies compared to non-stimulated cultures (*P* = 0.006 for 100 ng/mL; *P* = 0.015 for 200 ng/mL; Fig. 2J) and PMA alone (*P* = 0.0008 for PMA + 100 ng/mL; *P* = 0.009 for PMA + 200 ng/mL; Fig. 2J). Moreover, TIM-3^+^ NK cell frequency showed a dose-dependent response, decreasing under PMA at 0–20 ng/mL (*P* = 0.0003 for PMA, *P* = 0.013 for PMA + 10 ng/mL, *P* = 0.0002 for PMA + 20 ng/mL; Fig. 2L) but increasing at 100 ng/mL in the presence of PMA (*P <* 0.0001) compared to unstimulated conditions at these same doses and PMA alone (*P <* 0.01; Fig. 2L).

Collectively, these findings suggest that propranolol regulates NK cell phenotypes in a dose-dependent manner. Lower propranolol concentrations (0–50 ng/mL) appear to enhance PMA-induced effector functions, whereas higher concentrations (100–200 ng/mL) induce a shift toward a phenotype characterized by increased activation but reduced cytotoxic potential. This shift is marked by the upregulation of regulatory and exhaustion-associated markers, including KLRG1, LAG-3, and PD-1, which may contribute to the attenuation of inflammatory responses and a reprogramming of NK cell functional capacity.

#### Comparison of NK cell phenotypes and marker frequency between older untrained and trained groups in response to propranolol and inflammatory stimulation

**Fig. 3:**
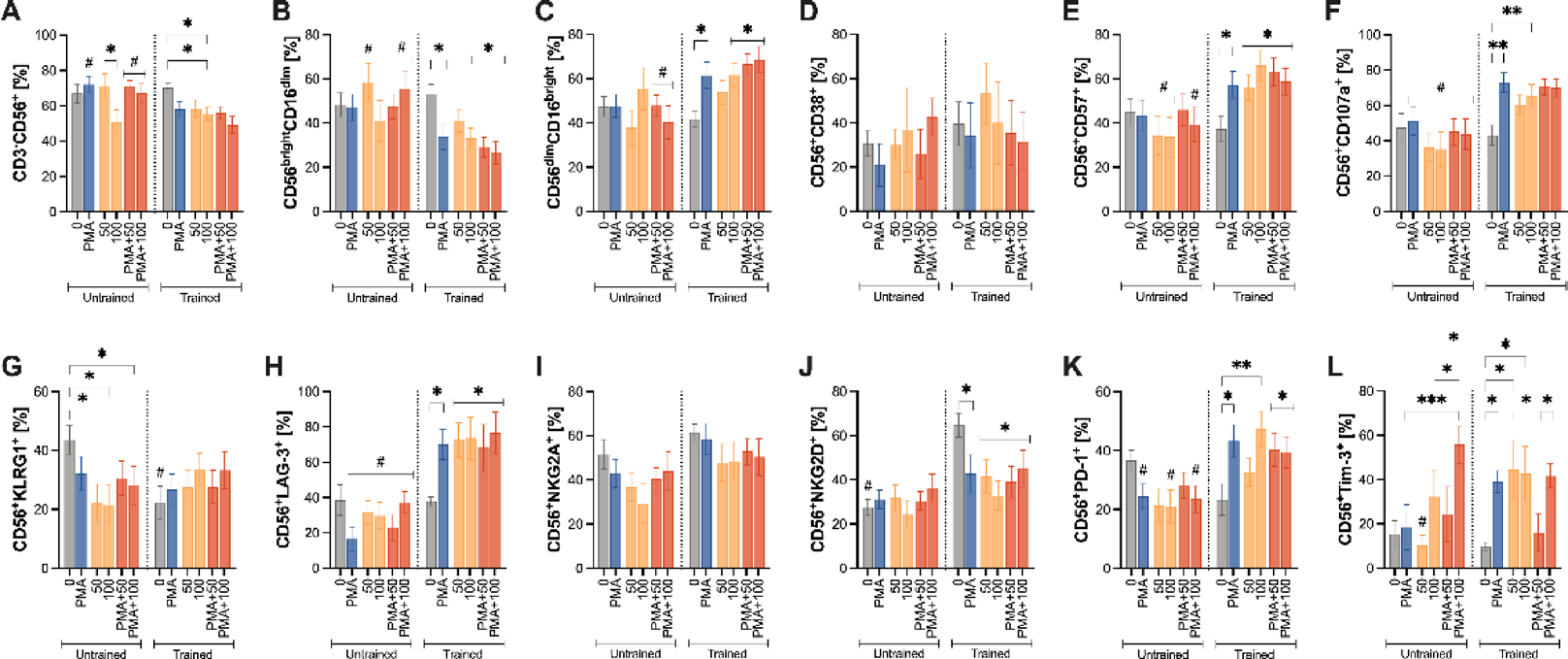
Differential effects of propranolol and inflammatory stimulation on NK-cell phenotype in untrained versus trained groups. Expanded NK cells were treated with propranolol (50 or 100 ng/mL) in the absence or presence of an inflammatory cocktail (PMA/ionomycin with brefeldin A and monensin). (**A**) Total NK cells (CD3⁻CD56^+^). (**B**,**C**) Cytotoxic (CD56^dim^CD16^bright^) and effector (CD56^bright^CD16^dim^) subset frequencies. (**D**–**F**) Expression of activation (CD38), differentiation (CD57), and degranulation (CD107a) markers. (**G**) Senescence marker KLRG1. (**H**–**J**) Regulatory receptors LAG-3, NKG2A, and NKG2D. (**K**,**L**) Immune-exhaustion markers PD-1 and TIM-3. Data are presented as mean ± SEM from *n* = 4–5 donors per group. * *P* < 0.05 versus untreated control; # *P* < 0.05 between untrained and trained at the same concentration (Bonferroni post hoc test following a significant interaction effect in two-way ANOVA). CD, cluster of differentiation; KLRG1, killer cell lectin-like receptor subfamily G member 1; LAG-3, lymphocyte-activation gene 3; NK, natural killer; NKG2A, natural killer group 2, member A; NKG2D, natural killer group 2, member D; PD-1, programmed death-1; PMA, phorbol 12-myristate 13-acetate; TIM-3, T-cell immunoglobulin and mucin-domain containing-3.

To investigate exercise-induced adaptations in NK cells, we compared expanded NK cells from older untrained and older trained individuals following treatment with propranolol (0, 50, or 100 ng/mL), PMA, and the combination of PMA + propranolol. Propranolol concentrations of 10, 20, and 200 ng/mL were excluded because they either yielded results similar to control (0 ng/mL propranolol) or were associated with reduced cell viability in the dose-response analysis (Fig. 1D). No significant stimulus × group interaction effects were observed for CD56^+^CD38^+^ (*P* = 0.389; Fig. 3D) and CD56^+^NKG2A+ (*P* = 0.827; Fig. 3I) cells (Suppl. Table 2).

In the trained group, 100 ng/mL propranolol significantly decreased NK cell (CD3⁻CD56^+^) frequency compared to control (*P* = 0.0003; Fig. 3A). Under PMA-stimulated conditions, the trained group showed lower NK cell frequencies at PMA (*P* = 0.042), PMA + 50 ng/mL propranolol (*P* = 0.029), and PMA + 100 ng/mL (*P* = 0.010) compared to the untrained group (Fig. 3A). Regarding NK cell subsets, the trained group showed reduced cytotoxic NK cells (Fig. 3B), but increased effector NK cells (Fig. 3C) under 100 ng/mL propranolol (*P* = 0.017 and *P* = 0.0162, respectively), PMA (*P* = 0.025 and *P* = 0.016), or PMA + 50 ng/mL (*P* = 0.0029 and *P* = 0.0008) or 100 ng/mL propranolol (*P* = 0.0003 and *P* = 0.0002) relative to control (Fig. 3B-C). Compared to the untrained group, the trained group had lower cytotoxic NK cells at 50 ng/mL propranolol (*P* = 0.039), PMA + 50 ng/mL (*P* = 0.032), or PMA + 100 ng/mL (*P* = 0.009; Fig. 3B), and higher effector cells with PMA + 50 ng/mL (*P* = 0.028) or PMA + 100 ng/mL (*P* = 0.001; Fig. 3C).

In the trained group, PMA (*P* = 0.018) and propranolol at 50 ng/mL and 100 ng/mL (*P* = 0.032; *P* < 0.0001, respectively) increased the frequency of CD57^+^ NK cells relative to control (Fig. 3E). Further increases were observed with PMA + 50 ng/mL (*P* = 0.0005) and PMA + 100 ng/mL (*P* = 0.007; Fig. 3E). The trained group also consistently showed higher CD57^+^ NK cell frequencies than the untrained group at 50 ng/mL propranolol (*P* = 0.026), 100 ng/mL propranolol (*P* = 0.001), and PMA + 100 ng/mL (*P* = 0.045; Fig. 3E). Similarly, in the trained group, 100 ng/mL propranolol (*P* = 0.003) and PMA (*P* < 0.0001) significantly increased the frequency of CD107a^+^ NK cells relative to control (Fig. 3F). Moreover, the trained group consistently had higher CD107a^+^ frequencies than the untrained group under 50 ng/mL propranolol (*P* = 0.013), 100 ng/mL propranolol (*P* = 0.002), PMA (*P* = 0.024), and PMA + 50 ng/mL (*P* = 0.008) or PMA + 100 ng/mL (*P* = 0.007; Fig. 3F).

In the untrained group, propranolol at 50 ng/mL and 100 ng/mL significantly reduced KLRG1^+^ frequency compared to control (*P* < 0.0001; Fig. 3G). The untrained group had a higher KLRG1^+^ NK cell frequency than the trained group at baseline (*P* = 0.014; Fig. 3G), suggesting a more senescent NK cell profile in the untrained cohort. Conversely, at baseline, the trained group had higher frequency of NKG2D^+^ NK cell than the untrained group (*P* = 0.0002; Fig. 3J). Within the trained group, NKG2D^+^ frequency decreased at 50 ng/mL propranolol (*P* = 0.002) or 100 ng/mL propranolol (*P* < 0.0001), PMA (*P* = 0.003), and PMA + 50 ng/mL (*P* = 0.0003) or PMA + 100 ng/mL (*P* = 0.013) relative to control (Fig. 3J). In the trained group, propranolol (*P* = 0.009 for 50 ng/mL; *P* = 0.007 for 100 ng/mL) and PMA (*P* = 0.02) each increased LAG-3^+^ NK cell frequency relative to control (Fig. 3H). Further increases were observed with PMA + 50 ng/mL (*P* = 0.032) or PMA + 100 ng/mL (*P* = 0.003; Fig. 3H). Compared to the untrained group, LAG-3^+^ NK cell frequency was higher in the trained group under propranolol and PMA-stimulated conditions (*P* = 0.002 for 50 ng/mL; *P* = 0.001 for 100 ng/mL; *P* = 0.0001 for PMA; *P* = 0.0008 for PMA + 50 ng/mL; P. 0.003 for PMA + 100 ng/mL; Fig. 3H).

In the untrained group, 100 ng/mL propranolol (*P* = 0.0001), PMA (*P* = 0.002), and PMA + 50 ng/mL or PMA + 100 ng/mL (*P* = 0.018 and *P* = 0.029, respectively) increased PD-1^+^ NK cell frequency relative to control (Fig. 3K). PD-1^+^ levels were also higher in the trained group than in the untrained group at 100 ng/mL propranolol (*P* = 0.007), PMA (*P* = 0.014), and PMA + 100 ng/mL (*P* = 0.036; Fig. 3K). For TIM-3^+^ NK cells, the trained group showed increased frequencies at 50 ng/mL propranolol (*P* = 0.0006), 100 ng/mL propranolol (*P* = 0.001), and PMA (*P* = 0.005) relative to control (Fig. 3L). In the untrained group, PMA + 100 ng/mL increased TIM-3^+^ NK cell frequency compared to PMA (*P* = 0.0002) and 100 ng/mL propranolol (*P* = 0.045; Fig. 3L). Additionally, at 50 ng/mL propranolol, trained individuals had a higher TIM-3^+^ NK cell frequency than untrained individuals (*P* = 0.011; Fig. 3L).

Overall, these data suggest that exercise training in older adults is associated with a more adaptive NK cell response to propranolol and inflammatory stimulation, characterized by enhanced effector functions (e.g., increased CD57^+^ and CD107a^+^ frequencies), reduced markers of senescence (e.g., lower baseline KLRG1^+^), and greater regulatory marker expression (e.g., LAG-3^+^, PD-1^+^, and TIM-3^+^).

### Energetic cell sensors: how do NK cells adapt to restrictive metabolic environments?

#### Rapamycin dose-response effects on NK cell phenotypes, activation, and regulatory markers

**Fig. 4:**
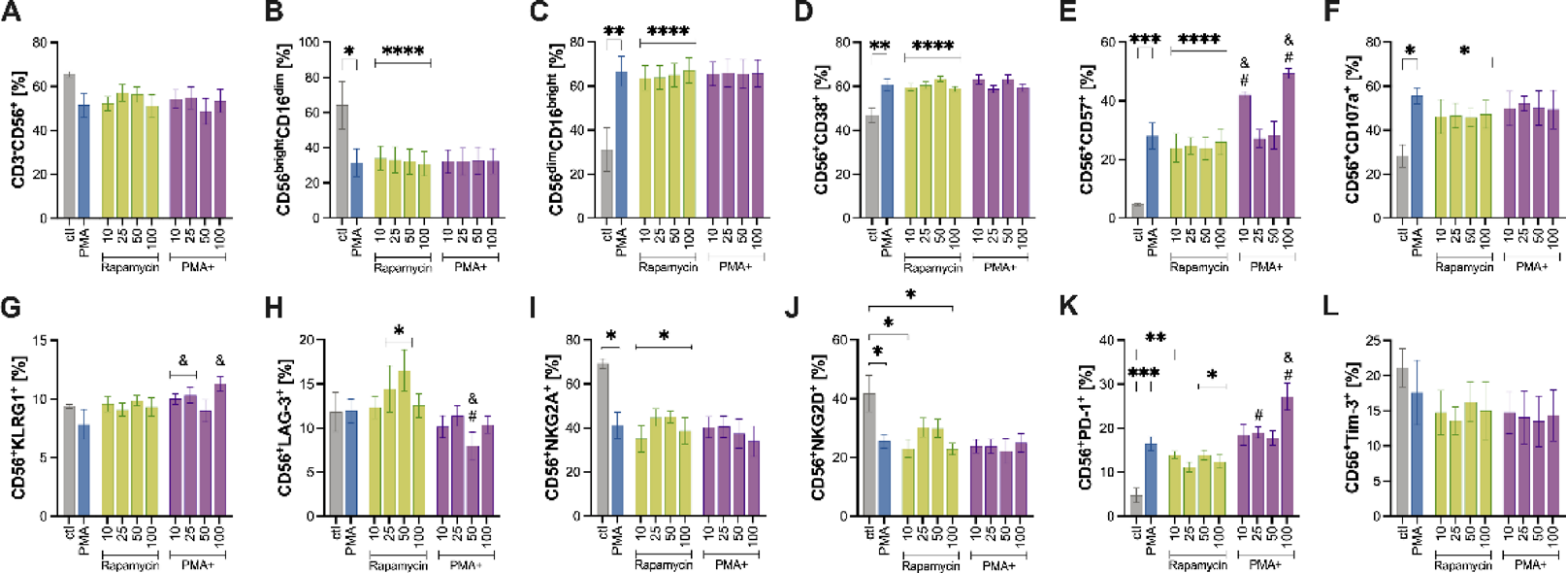
Dose-response effects of rapamycin on NK cells phenotype, activation, and regulatory receptor expression. NK cells expanded for 14 days were treated with increasing concentrations of rapamycin (0, 10, 25, 50, or 100 ng/mL) in the absence or presence of an inflammatory stimulus (PMA, ionomycin, brefeldin A, and monensin). (**A**) Total NK cells (CD3⁻CD56^+^). (**B**,**C**) Cytotoxic (CD56^dim^CD16^bright^) and effector (CD56^bright^CD16^dim^) subset. (**D**–**F**) Expression of activation (CD38), differentiation (CD57), and degranulation (CD107a) markers. (**G**) Senescence marker KLRG1. (**H**–**J**) Regulatory receptors LAG-3, NKG2A, and NKG2D. (**K**,**L**) Immune-exhaustion markers PD-1 and TIM-3. Data are presented as mean ± SEM from *n* = 4 untrained and *n* = 5 endurance-trained donors. * *P* < 0.05 versus untreated control; & *P* < 0.05 versus PMA; # *P* < 0.05 between PMA-stimulated and unstimulated at the same dose. All comparisons were determined using Bonferroni post hoc tests following a significant interaction effect detected by two-way ANOVA. CD, cluster of differentiation; KLRG1, killer cell lectin-like receptor subfamily G member 1; LAG-3, lymphocyte-activation gene 3; NK, natural killer; NKG2A, natural killer group 2, member A; NKG2D, natural killer group 2, member D; PD-1, programmed death-1; PMA, phorbol 12-myristate 13-acetate; TIM-3, T-cell immunoglobulin and mucin-domain containing-3.

To elucidate how NK cells adapt to restrictive metabolic environments, we investigate the effects of increasing rapamycin concentrations (10, 25, 50, and 100ng/mL) on NK cell phenotypes. Expanded NK cells were also cultured in the absence or presence of an inflammatory cocktail (PMA) to simulate inflammatory conditions (Suppl. Table 3). No significant stimulus × group interaction effects were observed for CD56^+^Tim3^+^ (*P* = 0.075; Fig. 4L; Suppl. Table 3).

PMA stimulation significantly reduced total NK cell frequency (*P* = 0.034; Fig. 4A). Moreover, both rapamycin (10–100 ng/mL) and PMA markedly decreased the proportion of cytotoxic NK cells (*P* < 0.0001 for all rapamycin doses; *P* = 0.0008 for PMA; Fig. 4B) while increasing effector NK cells (*P* < 0.0001 for all rapamycin doses; *P* = 0.0005 for PMA; Fig. 4C).

PMA stimulation alone significantly increased the frequency of CD38^+^ (*P* = 0.0006; Fig. 4D), CD57^+^ (*P* = 0.0001; Fig. 4E), and CD107a^+^ (*P* = 0.006; Fig. 4F), and reduced NKG2A+ (*P* = 0.001; Fig. 4I) NK cells compared to control (0 ng/mL rapamycin). Rapamycin alone at 10–100 ng/mL also elevated CD38+, CD57+, and CD107a + NK cell frequencies (P *<* 0.05 for all; Fig. 4D-F), while reduced NKG2A + NK cells (*P <* 0.0001 for all; Fig. 4I). When combined with PMA, rapamycin at 10 ng/mL and 100 ng/mL further increased CD57 + NK cell frequency compared to rapamycin alone at the same concentrations (*P* = 0.002 and *P =* 0.0001, respectively) and PMA alone (*P* = 0.002 for PMA + 10 ng/mL and *P <* 0.0001 for PMA + 100 ng/mL), suggesting a possible synergistic effect on this maturation/activation marker (Fig. 4E). Likewise, PMA in combination with 10 ng/mL (*P* = 0.002), 25 ng/mL (*P* = 0.0005) and 100 ng/mL rapamycin (*P <* 0.0001) increased KLRG1 + NK cell frequency compared to PMA alone (Fig. 4G), indicating that rapamycin can potentiate PMA on senescence-associated pathways. Surprisingly, rapamycin at 10 ng/mL and 100 ng/mL (*P* = 0.001 for both) and PMA (*P* = 0.003) reduced the NKG2D + NK cells frequency (Fig. 4J).

Regarding PD-1, PMA (*P <* 0.0001) and rapamycin treatment at 10 ng/mL (*P* = 0.005), 50 ng/mL (*P* = 0.005), and 100 ng/mL (*P* = 0.020) increased the frequency of PD-1 + NK cells relative to control (Fig. 4K). Moreover, PMA + 100 ng/mL rapamycin increased PD-1 + NK cell frequency compared to either treatment alone (*P <* 0.0001 for 100 ng/mL rapamycin, *P* = 0.001 for PMA; Fig. 4K). In contrast, LAG-3 upregulation by rapamycin was blunted by PMA at 50 ng/mL rapamycin. Specifically, the frequency of LAG-3 + was significantly lower with PMA + 50 ng/mL than 50 ng/mL rapamycin (*P* = 0.005) and PMA alone (*P* = 0.003; Fig. 4H).

Collectively, these results show that rapamycin dose-dependently reshapes NK cell phenotypes, reducing cytotoxic NK cells while increasing effector subsets. Rapamycin enhances activation markers (CD38, CD57, CD107a) and senescence marker (KLRG1), particularly when combined with PMA at 10 and 100 ng/mL, suggesting synergistic effects on maturation. Conversely, rapamycin reduces NKG2D, indicating diminished cytotoxic potential, while promoting PD-1 expression, especially at high doses with PMA, reflecting exhaustion-like features.

#### Comparison of NK cell phenotypes and marker frequency between untrained and trained groups in response to rapamycin and inflammatory stimulation

**Fig. 5:**
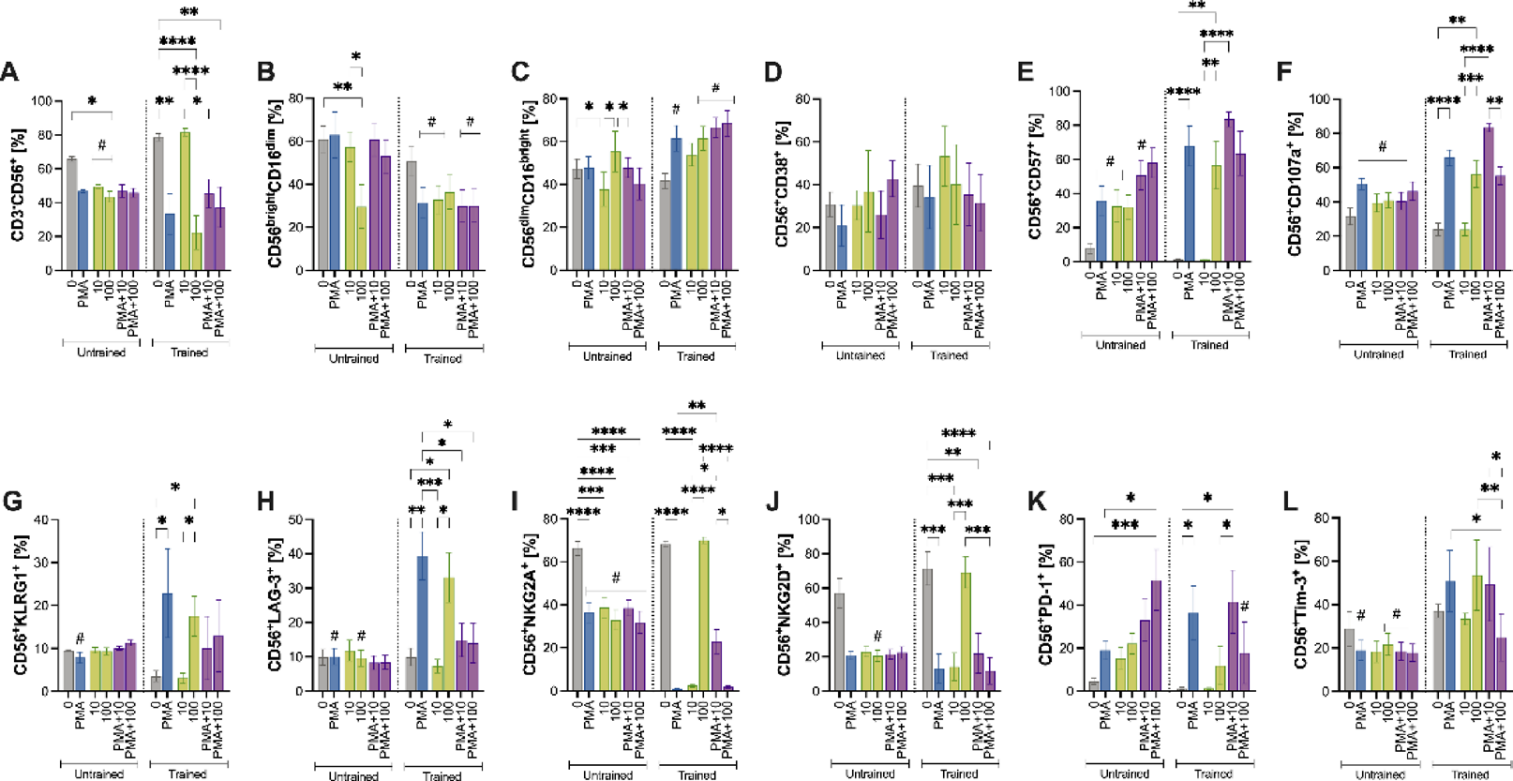
Differential effects of propranolol and inflammatory stimulation on NK-cell phenotype in untrained versus trained older adults. Expanded NK cells were treated with rapamycin (10 or 100 ng/mL) in the absence or presence of an inflammatory cocktail (PMA/ionomycin with brefeldin A and monensin). (**A**) Total NK cells (CD3⁻CD56^+^). (**B**,**C**) Cytotoxic (CD56^dim^CD16^bright^) and effector (CD56^bright^CD16^dim^) subset frequencies. (**D**–**F**) Expression of activation (CD38), differentiation (CD57), and degranulation (CD107a) markers. (**G**) Senescence marker KLRG1. (**H**–**J**) Regulatory receptors LAG-3, NKG2A, and NKG2D. (**K**–**L**) Immune-exhaustion markers PD-1 and TIM-3. Data are presented as mean ± SEM from *n* = 4–5 donors per group. * *P* < 0.05 versus untreated control; # *P* < 0.05 between untrained and trained at the same concentration (Bonferroni post hoc test following a significant interaction effect in two-way ANOVA). CD, cluster of differentiation; KLRG1, killer cell lectin-like receptor subfamily G member 1; LAG-3, lymphocyte-activation gene 3; NK, natural killer; NKG2A, natural killer group 2, member A; NKG2D, natural killer group 2, member D; PD-1, programmed death-1; PMA, phorbol 12-myristate 13-acetate; TIM-3, T-cell immunoglobulin and mucin-domain containing-3.

We next compared NK cell phenotypes between untrained versus trained older adults with rapamycin (10 ng/mL and 100 ng/mL) and/or PMA stimulation (Fig. 5). Based on dose-response findings, 25 ng/mL and 50 ng/mL rapamycin were excluded because they either produced similar results or impacted cell viability (Fig. 1E). No significant stimulus × group interaction effects were observed for CD56^+^CD38^+^ (*P* = 0.956; Fig. 5D) (Suppl. Table 4).

In the untrained group, 100 ng/mL rapamycin reduced NK cell (CD3⁻CD56^+^) frequency relative to control (*P* = 0.034; Fig. 5A). In the trained group, NK cell frequency was significantly lower under both PMA stimulation and 100 ng/mL rapamycin compared to control (*P* < 0.0001), with 100 ng/mL rapamycin further decreasing frequency compared to 10 ng/mL (*P* < 0.0001) and relative to 100 ng/mL rapamycin at the untrained group (*P* = 0.044; Fig. 5A). Notably, at 10 ng/mL rapamycin, the trained group showed higher NK cell frequencies than the untrained group (*P* = 0.008; Fig. 5A).

For the cytotoxic subset (CD56^bright^CD16^dim^), the untrained group showed decreased frequencies at 100 ng/mL rapamycin compared to control (*P =* 0.0001; Fig. 5B). Compared to the trained group, cytotoxic NK cell frequency was higher in the untrained group at 10 ng/mL rapamycin (*P* = 0.006), PMA (*P* = 0.032), PMA + 10 ng/mL (*P* = 0.007), and PMA + 100 ng/mL (*P* = 0.044; Fig. 5B). For the effector NK subset (CD56^dim^CD16^bright^), the untrained group showed increased frequencies at 10 ng/mL rapamycin compared to control (*P =* 0.046), 100 ng/mL (*P =* 0.025), and PMA + 10 ng/mL rapamycin (*P =* 0.018; Fig. 5C), suggesting that partial mTOR inhibition may support NK cell activation. Within the trained group, effector NK cell frequency increased only when PMA was combined with rapamycin at PMA + 10 ng/mL and PMA + 100 ng/mL (*P =* 0.027 and *P =* 0.031, respectively) relative to control (Fig. 5C). Compared to the untrained group, the trained group exhibited higher effector NK cell frequencies at 100 ng/mL rapamycin (*P* = 0.010), PMA (*P* = 0.002), PMA + 10 ng/mL (*P* = 0.006) and PMA + 100 ng/mL (*P* = 0.018; Fig. 5C).

Regarding CD57^+^ NK cells, the trained group showed significant increases with PMA and 100 ng/mL rapamycin versus control, and these levels remained elevated under PMA + 10 ng/mL rapamycin or PMA + 100 ng/mL rapamycin (*P <* 0.0001 for all; Fig. 5E). In the untrained group, PMA (*P* = 0.032), PMA + 10 ng/mL rapamycin (*P* = 0.0002), or PMA + 100 ng/mL rapamycin (*P* < 0.0001) also increased CD57 + cell frequency (Fig. 5E). Between groups, the trained group had a lower CD57 + frequency at 10 ng/mL rapamycin (*P* = 0.015) but higher frequencies under PMA (*P* = 0.008) and PMA + 10 ng/mL rapamycin (*P* = 0.012) compared to the untrained group (Fig. 5E).

In the trained group, treatment with PMA or 100 ng/mL rapamycin significantly increased the frequency of CD107a^+^ NK cells compared to control (*P* < 0.0001 for both; Fig. 5F). Notably, combining PMA with 10 ng/mL rapamycin further elevated CD107a expression relative to either 10 ng/mL rapamycin or PMA combined with 100 ng/mL rapamycin (*P* < 0.0001 for both comparisons; Fig. 5F). Overall, the trained group exhibited enhanced degranulation capacity, with higher CD107a^+^ frequencies under PMA stimulation (*P* = 0.034 for PMA alone and *P* < 0.0001 for PMA + 10 ng/mL rapamycin) and rapamycin at 100 ng/mL (*P* = 0.033; Fig. 5F) compared to the untrained group. Conversely, under 10 ng/mL rapamycin, the trained group showed a lower CD107a^+^ frequency than the untrained group (*P* = 0.002; Fig. 5F).

In the trained group, KLRG1 + NK cells frequency increased with PMA (*P* = 0.002) and 100 ng/mL rapamycin (*P* = 0.036) relative to control (Fig. 5G). Similarly, LAG-3 + NK cells were elevated under PMA and 100 ng/mL rapamycin (*P <* 0.0001 for both) compared to control (Fig. 5H). Notably, 100 ng/mL rapamycin induced a higher LAG-3 + frequency than 10 ng/mL rapamycin (*P <* 0.0001) or PMA + 100 ng/mL rapamycin (*P* = 0.0001; Fig. 5H). Conversely, combining PMA with either 10 ng/mL or 100 ng/mL rapamycin reduced LAG-3^+^ frequency relative to PMA alone (*P <* 0.0001). Overall, the trained group exhibited higher LAG-3^+^ frequencies under PMA (*P <* 0.0001) and 100 ng/mL rapamycin (*P* = 0.0005) than the untrained group (Fig. 5H).

In both groups, NKG2A^+^ NK cell frequency decreased under all tested conditions relative to 0 ng/mL, except with 100 ng/mL rapamycin in the trained group (*P >* 0.999; Fig. 5I). Within the trained group, NKG2A + NK cells were lower than in untrained group under 100 ng/mL rapamycin (*P <* 0.0001), PMA (*P <* 0.0001), PMA + 10 ng/mL rapamycin (*P* = 0.004) and PMA + 100 ng/mL rapamycin (*P <* 0.0001), but higher at 10 ng/mL rapamycin (*P <* 0.0001; Fig. 5I). Similarly, both groups showed reduced frequencies of NKG2D^+^ NK cells under 100 ng/mL rapamycin, PMA, PMA + 10 ng/mL rapamycin, and PMA + 100 ng/mL rapamycin (*P <* 0.0001 vs. control), except the trained group at 100 ng/mL rapamycin, which remained similar to control (*P >* 0.999; Fig. 5J). Under 100 ng/mL rapamycin, the trained group had a higher NKG2D + frequency than the untrained group (*P <* 0.0001; Fig. 5J).

PMA stimulation alone and in combination with 10 ng/mL rapamycin increased PD-1 + NK cell frequency in the trained group compared to control (*P* = 0.012 and *P* = 0.003, respectively; Fig. 5K). However, when PMA was combined with 100 ng/mL rapamycin, PD-1 + NK cell frequency increased relative to control in the untrained group (*P* = 0.023) and was higher compared to the trained group at this same concentration (*P* = 0.016; Fig. 5K). Within the trained group, TIM-3 + NK cell frequency was lower under PMA + 100 ng/mL rapamycin compared to PMA (*P* = 0.018), PMA + 10 ng/mL rapamycin (*P* = 0.029) or 100 ng/mL rapamycin alone (*P* = 0.007; Fig. 5L). In the trained group, TIM-3 + frequency was higher compared to the untrained group under 100 ng/mL rapamycin (*P* = 0.024), PMA (*P* = 0.024), and PMA + 10 ng/mL rapamycin (*P* = 0.028; Fig. 5L).

Overall, these data show that the endurance-trained group exhibits stronger NK activity under PMA stimulation—particularly for CD107a and CD57—while lower-dose rapamycin (10 ng/mL) largely preserves NK function (e.g., maintaining cytotoxic subsets and degranulation). In contrast, higher-dose rapamycin (100 ng/mL) promotes markers of terminal differentiation or exhaustion (e.g., CD57, LAG-3) and can dampen certain activating pathways (e.g., NKG2D) in the trained group.

### Trained individuals exhibit higher oxygen consumption rates and a preference for aerobic metabolism compared to age-matched untrained individuals

**Fig. 6:**
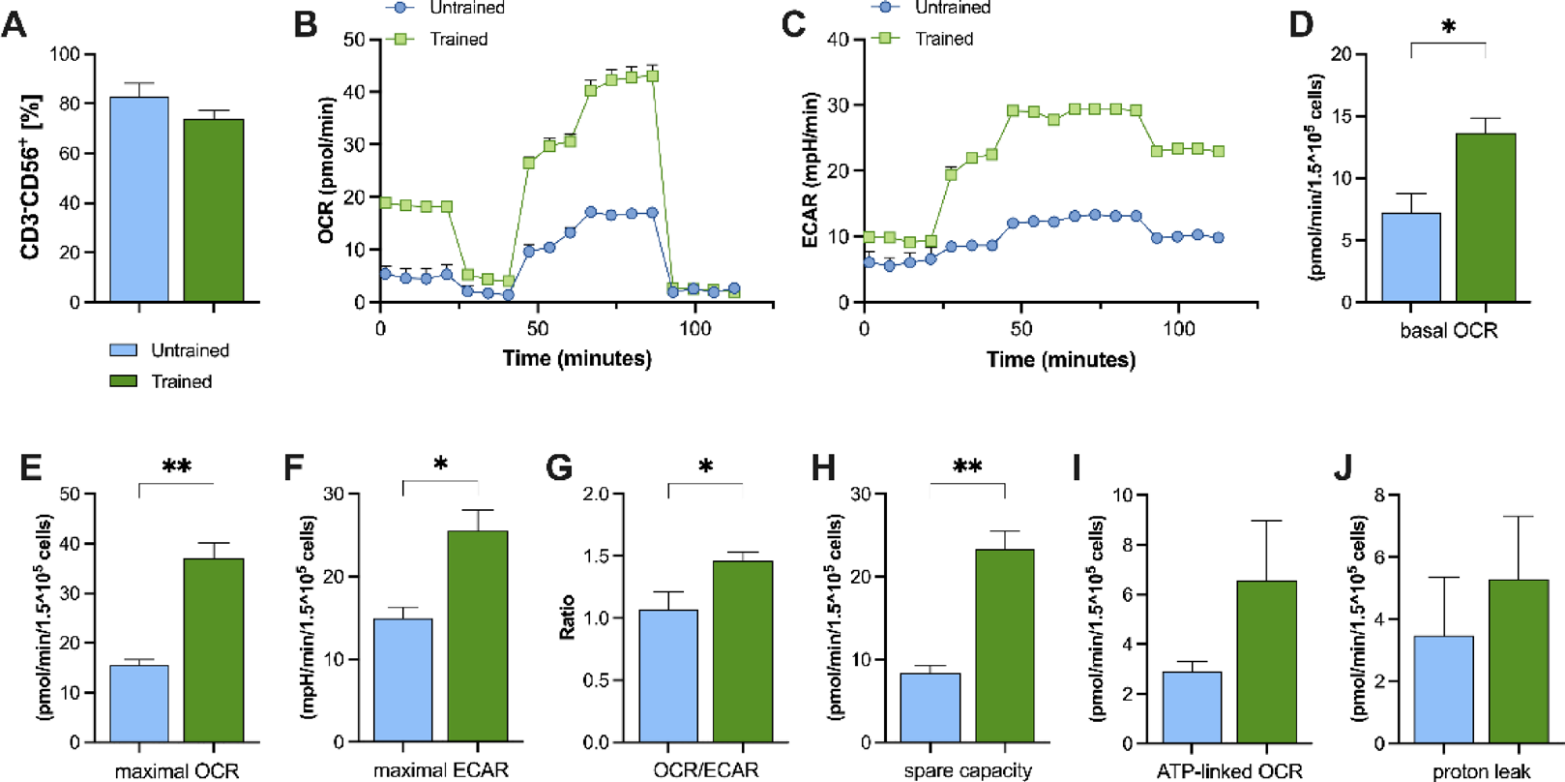
Enhanced oxidative metabolism in NK cells from trained versus untrained older adults. (**A**) Frequency of isolated NK cells (CD3⁻CD56^+^) in peripheral blood. (**B**,**C**) Representative Seahorse metabolic flux traces showing OCR and ECAR for NK cells from untrained (blue circles) and endurance-trained (green squares) donors. (**D**) Basal OCR. (**E**) Maximal OCR, measured after FCCP injection. (**F**) Spare respiratory capacity (maximal OCR – basal OCR). (**G**) Maximal ECAR. (**H**) OCR/ECAR ratio, indicating relative reliance on oxidative versus glycolytic metabolism. (**I**) ATP-linked OCR (basal OCR – oligomycin-inhibited OCR). (**J**) Proton leak (oligomycin‐inhibited OCR – non-mitochondrial respiration). Data are presented as mean ± SEM from *n* = 4 untrained and *n* = 5 endurance-trained donors. * *P* < 0.05 versus untreated control (Mann-Whitney U test). OCR, oxygen‐consumption rate; ECAR, extracellular acidification rate; FCCP, carbonyl cyanide-p-trifluoromethoxyphenylhydrazone.

NK cells were isolated from fresh peripheral blood samples using negative selection enrichment, and metabolic activity was subsequently assessed through respirometry analysis (Fig. 6). Purity of isolated CD3^−^CD56^+^ ranged from 70 to 85%, as determined by flow cytometry (Fig. 6A). Following Seahorse metabolic flux traces for OCR (Fig. 6B) and ECAR (Fig. 6C) in NK cells, endurance-trained individuals exhibited significantly higher basal OCR compared to the untrained group (*P* = 0.036; Fig. 6D), reflecting an enhanced ability to meet basic energy demands. Moreover, NK cells from endurance-trained individuals exhibited a significantly higher maximal OCR upon FCCP injection (*P* = 0.002; Fig. 6E).

Additionally, endurance-trained individuals showed a higher spare respiratory capacity (*P =* 0.002; Fig. 6F). Trained individuals also showed a higher maximal extracellular acidification rate (ECAR; *P =* 0.024; Fig. 6G). Notably, the OCR/ECAR ratio was greater in the trained group (*P =* 0.029; Fig. 6H), indicating a preference for aerobic metabolism. No significant differences in ATP-linked OCR were found between the groups (*P =* 0.300; Fig. 6I). Both groups also exhibited comparable proton leak (*P =* 0.786; Fig. 6J).

## Discussion

The metabolism of immune cells is closely linked to their phenotype and cytokine production, with various factors such as hormonal response, aging, and physical fitness levels influencing this relationship^25–27^. In this pilot study, we provide evidence that energy sensors—particularly those involving the mTOR pathway and adrenergic signaling—drive significant modifications in NK cell phenotype and metabolism. Furthermore, exercise training appears to promote more efficient aerobic metabolism in NK cells.

We examined the impact of propranolol and PMA on NK cell phenotypes in trained versus untrained older adults. Notably, trained individuals consistently exhibit higher frequencies of CD107a^+^ and CD57^+^ NK cells under PMA stimulation, indicative of superior degranulation and cytotoxic capacity, as well as a more robust activation profile marked by higher NKG2D^+^ expression. In contrast, the untrained group had increased frequencies of KLRG1 + NK cells—a marker of senescent NK cells—suggesting that aging-associated senescence may be exacerbated by a lack of regular exercise.

At lower propranolol doses, propranolol and PMA acted synergistically to enhance effector markers such as CD107a and CD57. However, at higher doses (100 ng/mL), propranolol partially reduced NK cell activation in both groups. Nevertheless, even under β-adrenergic blockade, the trained group maintained superior effector responses and exhibited a more controlled expression of inhibitory receptors, such as PD-1, LAG-3, and TIM-3. These findings support previous evidence that regular exercise improves NK cell degranulation and cytotoxicity^28^ by potentially promoting adaptative β2-adrenergic signaling pathways.

Our results further reveal that while both propranolol and PMA decreased the frequency of NKG2D + NK cells in a dose-dependent manner, trained individuals better preserved NKG2D expression upon PMA stimulation. One plausible explanation is that the robust activation signal provide by PMA—administrated along with ionomycin, brefeldin A, and monensin—leads to transient internalization or temporarily downregulation to prevent overactivation or cytotoxic damage^29^. Additionally, secretion inhibitors such as brefeldin A and monensin may interfere with receptor recycling, further reducing detectable NKG2D on the cell surface. Thus, the observed decrease in NKG2D under PMA stimulation likely reflects a controlled, physiological process that fine-tunes NK cell activation during intense inflammatory signaling in trained older adults.

The elevated CD57^+^ NK cells seen in the trained group underscores the development of a more mature NK cell phenotype^30^. Concurrently, the higher expression of inhibitory markers such as PD-1 and TIM-3 in trained individuals may serve as protective checkpoints to prevent excessive inflammation and autoreactivity^31,32^. In line with Bigley et al. (2015)^33^these adaptations suggest that regular training primes NK cells for both rapid activation and timely regulatory control.

Our rapamycin experiments further demonstrate that NK cell responses are regulated in a dose-dependent manner by mTOR inhibition. At lower doses (10 ng/mL), the trained group often maintained—or even enhanced—key NK cell functions (e.g., CD107a^+^ degranulation), especially when combined with PMA. In contrast, higher doses (100 ng/mL) induced a shift toward more regulatory phenotype, as evidenced by increased LAG-3^+^ and TIM-3^+^ NK cells. These findings indicate that regular physical exercise may help maintain a flexible mTOR-mediated metabolic state, allowing NK cells to balance activation and inhibition effectively despite partial mTOR blockade. This is consistent with the critical role of mTOR signaling in orchestrating the metabolic transitions required for NK cell activation, as mTORC1 activity drives the switch from oxidative phosphorylation to glycolysis during effector responses^19,34,35^. Moreover, our data align with prior studies demonstrating that mTORC1 inhibition through genetic deletion reduces NK cell maturation and function, including decreases in the number of mature CD11b^+^CD27^+^ NK cells and impairments in effector molecule production such as perforin and granzyme, as well as glucose uptake, which are critical for NK cell-mediated killing^36^.

Interestingly, under high-dose rapamycin, trained individuals showed higher frequencies of CD57^+^ NK cells compared to untrained subjects, that chronic exercise may fine-tune NK cell maturation, promoting a state in which cells are primed for rapid and potent responses upon inflammatory stimulation^30^. In contrast, the untrained group exhibited more pronounced markers of senescence (e.g., KLRG1) and were more susceptible to propranolol-induced reductions in NK cell frequency, reinforcing the concept that chronic exercise mitigates immunosenescence^3^.

The cumulative evidence from our study underscores that benefits of regular exercise on NK cell function are mediated by repeated acute exercise bouts, which cumulatively enhance both metabolic efficiency and immune regulation^37^. Acute exercise mobilizes NK cells via adrenaline-stimulated β2-adrenergic receptors^38,39^leading to rapid increases in cytotoxicity and migration^40,41^. Repetitive adrenergic stimulation, when coupled with adequate recovery, appears to promote lasting adaptations that preserve NK cell functionality under both inflammatory and inhibitory conditions^42^. Studies on exercise training have further demonstrated that rhythmic adrenergic stimulation positively impacts NK cell function, promoting both activation and immune regulation without the overstimulation seen in chronic stress^25,26,43,44^.

Finally, our OCR experiments revealed that NK cells from endurance-trained individuals exhibited significantly higher basal and maximal OCR, suggesting enhanced metabolism and mitochondrial respiration^45^. The higher OCR/ECAR ratio in the trained group indicates a greater reliance on oxidative phosphorylation over glycolysis for energy production under resting conditions. This suggests an aerobic metabolic preference within the limits of substrate availability in intact cells. Aerobic metabolism is crucial for meeting the high energy demands during NK cell activation^46,47^. Although ATP-linked respiration levels were comparable between groups, the improved metabolic parameters in trained individuals likely contribute to their resilience against pharmacological inhibition and stress-induced immune suppression^48^. Moreover, the comparable levels of proton leak between the groups would indicate that there is no additional mitochondrial damage in trained individuals, suggesting that their increased metabolic activity is not accompanied by higher mitochondrial dysfunction or inefficiency^49^.

A limitation of our study is the small sample size. Given this limitation, we consider our investigation as a pilot study. Using a comprehensive experimental design, which integrates cellular phenotyping, functional assays, and metabolic analyses, our findings provide directions for larger-scale studies to validate and further explore the mechanistic underpinnings of exercise-induced adaptations in NK cell activity. Moreover, NK cells were evaluated following in vitro expansion, without direct comparison to freshly isolated NK cells. Although the ImmunoCult™ NK Cell Expansion Kit supports robust proliferation while preserving key NK cell functions, in vitro culture conditions may still influence activation status, receptor expression, and metabolic profiles. Future studies should include parallel analysis of freshly isolated NK cells to validate the intrinsic differences observed between trained and untrained individuals and better delineate culture-induced effects.

## Conclusion

This study reveals that older adults with a history of endurance training exhibit a more functional, metabolically flexible NK cell phenotype compared to untrained counterparts. NK cells from endurance-trained individuals show enhanced effector functions, including higher degranulation (CD107a) and maturation (CD57) markers, lower senescence-associated KLRG1 expression, and greater metabolic flexibility characterized by increased oxidative phosphorylation and spare respiratory capacity. Furthermore, under pharmacologic stressors such as adrenergic blockade (propranolol) and mTOR inhibition (rapamycin), NK cells from trained individuals maintain superior functional profiles, including preserved cytotoxicity and greater adaptability to inflammatory conditions. These findings suggest that long-term endurance training is associated with protective immunometabolic adaptations in NK cells, potentially contributing to healthier immune aging.

## Methods

The medical ethics committee of the Justus-Liebig-University Giessen approved this study (AZ 100/20). All experimental procedures were performed according to the Declaration of Helsinki and all participants gave written informed consent before enrolment.

### Participants

Older male participants were recruited and allocated into untrained (*n* = 4; 64.3 ± 3.3 years) or endurance-trained (*n* = 5; 63.6 ± 2.1 years) groups. All participants were healthy older adults recruited as part of the “Giessen Immunaging” study^50^. Inclusion criteria comprised age > 55 years and absence of acute illness, including infections or injuries. Exclusion criteria comprised: excessive alcohol consumption (> 2 drinks/day), smoking, BMI < 18.5 kg/m^2^, prior history of myocardial infarction or cardiac disease, apoplexy, central or peripheral neuropathy, obstructive lung disease, metabolic diseases (e.g., type 1 or type 2 diabetes), systemic inflammatory or neoplastic diseases (e.g., cancer, arthritis, hepatitis, HIV, autoimmune conditions), and use of any medication affecting the immune system within the 12 weeks preceding the study.

Participants were categorized as endurance-trained or untrained using a structured internal questionnaire and cohort-ranked VO_2peak_ values. The questionnaire identified endurance-trained individuals as those who had ever competed in a sport and who were still actively engaged in that discipline—or who continued to practice it regularly as a hobby—and who reported following a structured, plan-based training regimen. These participants also displayed the highest VO_2peak_ values (43.2 ± 4.0 mL/kg/min) within the cohort. Conversely, the untrained group consisted of participants with no history of competitive sport or structured training and with the lowest cohort VO_2peak_ values (25.0 ± 3.6 mL/kg/min; Table 1).

### Cardiopulmonary exercise testing

All participants performed a ramp-incremental test to volitional exhaustion on an electronically braked cycle ergometer (Excalibur Sport^®^, Lode, The Netherlands). Two ramp protocols were used, depending on the participant’s training status, to ensure attainment of maximal workload within a 15-minute protocol. After a 3-minute warm-up at 0 W, untrained subjects followed a two-phase ramp (start at 50 W, + 25 W every 3 min; from 100 W onward, + 25 W every 2 min). Trained subjects began at 50 W and increased by 50 W every 3 min. Tests continued until one of the following exhaustion criteria was met: (a) Subjective exhaustion or intense dyspnea reported by the participant; (b) Achievement of ≥ 85% age-predicted maximal heart rate; (c) Peak respiratory exchange ratio (RER) > 1.1; c) VO_2_ plateau despite increasing workload. Ventilatory and gas exchange data were acquired breath‐by‐breath (Metalyzer 3-B system, Cortex, Germany). VO_2peak_ was defined as the average VO_2_ over the final 30 s of the test. This protocol has been described in detail elsewhere^50^.

### Cell culture assays

Venous fasting blood samples were taken from each participant between the hours of 06:30–08:30 for isolation of peripheral blood mononuclear cells (PBMCs) and further analysis. PBMCs were isolated by Ficoll-Paque density-gradient centrifugation and immediately seeded into a feeder-free, serum-free expansion system (ImmunoCult™ NK Cell Expansion Kit, STEMCELL Technologies, Vancouver, CA). Cells were cultured for 14 days according to the manufacturer’s protocol. On day 0, day 7, and after the 14-day expansion period, cells were washed and stained with anti-CD3 and anti-CD56 antibodies to phenotype the expanded NK cell population (Fig. 1A-B). Cell viability was assessed using Zombie Aqua™ (BioLegend, San Diego, CA) viability dye (Fig. 1C). In vitro expansion was essential to obtain the large NK cell numbers required for the extensive functional and metabolic assays performed across multiple treatment conditions. To ensure that the ex vivo expansion process preserved the key phenotypic characteristics of NK cells, we compared freshly isolated NK cells (Fig. 1F) with NK cells expanded for 14 days (Fig. 1A, third panel). Phenotypic characterization demonstrated that the frequencies of CD56+ (*P* = 0.190), CD56^dim^CD16^bright^ (*P* = 0.190), and CD56^bright^CD16^dim^ (*P* = 0.111) NK cell subsets remained largely comparable between freshly isolated and expanded cells (Fig. 1G). Minor, non-significant differences were observed in the frequency of CD107a+ (*P* = 0.063), CD57+ (*P* = 0.057), KLRG1 (*P* = 0.400), NKG2A (*P* = 0.190), and NKG2D (*P* = 0.730), which are expected due to the proliferation dynamics inherent to ex vivo expansion.

Following expansion, cells were washed again and resuspended in RPMI 1640 medium supplemented with 10% fetal bovine serum. The resuspended cells were then plated in flat-bottom 24-well plates and subjected to a 48-hour stimulation under the following conditions: (1) Propranolol: a nonselective β-ARs antagonist that targets both β1- and β2-ARs; (2) Rapamycin: an inhibitor of the mTORC1 pathway; (3) Cell stimulation cocktail: a combination of phorbol 12-myristate 13-acetate (PMA), ionomycin, brefeldin A, and monensin (eBioscience™ Cell Stimulation Cocktail, Thermo Fisher Scientific, Waltham, MA).

Following the manufacturer’s protocol, the PMA-containing stimulation cocktail was added to propranolol- or rapamycin-treated wells at a final 1:500 dilution to induce cell activation. After stimulation, the functional responses of the cells were assessed through cytokine release assays and flow cytometry. For control conditions, cells were treated with 0 ng/mL propranolol and 0 ng/mL rapamycin, serving as the baseline, with or without the addition of PMA to compare unstimulated and stimulated responses. All assays were performed in triplicate.

### Drug preparation

Propranolol hydrochloride (Cat. No. H26645.06; Thermo Fisher Scientific, Waltham, MA) was initially dissolved in dimethyl sulfoxide (DMSO) (Tocris, Oakville, Ontario, CA) to prepare a 50 mM stock solution. Prior to each experiment, the stock solution was freshly diluted in RPMI 1640 medium to achieve the required concentrations as specified for each assay. Rapamycin (Sigma-Aldrich, St. Louis, MO) was reconstituted in DMSO to generate a 10 mM stock solution. Similarly to propranolol, the rapamycin stock was freshly diluted in RPMI 1640 medium to the desired concentrations immediately before use in each experimental setup.

Expanded NK cells were cultured at 5 × 10·5 cells/mL with propranolol (0, 20, 50, 100, or 200 ng/mL) or rapamycin (0, 10, 25, 50, or 100 ng/mL) in the presence or absence of the cell stimulation cocktail. These ranges were selected to encompass clinically relevant plasma concentrations and to enable a dose-response analysis of NK-cell function. Therapeutic dosing of propranolol typically yields steady-state plasma levels between ~ 50 and 200 ng/mL; therefore, the chosen concentrations reproduce physiologically attainable exposure while maintaining cell viability. In-vitro studies have also demonstrated propranolol’s biological activity across this window^51^. Rapamycin concentrations were based on therapeutic exposure and prior work showing that 10–100 ng/mL rapamycin effectively inhibits NK cell proliferation and function in vitro^52^without inducing major cytotoxicity.

### NK cell phenotyping

After 48 h of incubation, cells were harvested from the plate and transferred to FACS tubes. The cells were then washed, resuspended in PBS, and immediately acquired in a CytoFLEX S and Kaluza analysis software 2.1 (Beckman Coulter, Indianapolis, IN). For acquisition initially, 50,000 to 200,000 events, corresponding to all nucleated cells present in the sample, were collected, and information was stored. Natural killer (NK) cells were identified as CD3⁻CD56^+^ lymphocytes. Two major NK cell subsets were defined based on the expression of CD56 and CD16: CD56^dim^CD16^bright^, representing the predominant cytotoxic NK cell subset, which is specialized in direct target cell lysis, and CD56^bright^CD16^dim/−^, representing the cytokine-producing or immunoregulatory NK cell subset, characterized by robust cytokine secretion and limited cytotoxicity. The frequency of terminally differentiated NK cells was assessed by the expression of CD57. NK cells were further characterized by the expression of markers related to functional states: Exhaustion markers: PD-1 (Programmed Cell Death Protein 1), TIM-3 (T-cell immunoglobulin and mucin-domain containing-3), and LAG-3 (Lymphocyte-activation gene 3); Senescence marker: KLRG1 (Killer cell lectin-like receptor subfamily G member 1); Inhibitory receptor: NKG2A (natural killer group 2, member A), which delivers inhibitory signals upon ligand engagement; Activating receptor: NKG2D, (natural killer group 2, member D), which directly triggers cytotoxic responses upon binding stress-induced ligands. Additionally, the expression of CD38 was evaluated as a marker associated with NK cell activation, metabolic regulation, and immunomodulatory functions.

Data were analyzed using CytExpert Acquisition and Analysis Software. A 7-panel phenotyping analysis (BioLegend, San Diego, CA) was as follows:

**Table:** 

Excitation laser	Detector/filter	Conjugates	Panel 1	Panel 2	Panel 3
Blue	488 nm	FITC	CD3	CD3	CD3
		PE	PD-1	NKG2A	TIM-3
		PerCP-Cy5.5	CD57	Cd107a	CD38
Red	633 nm	APC	KLRG1	NKG2D	LAG-3
		APC/Fire 750	CD16	CD16	CD16
Violet	405 nm	BV421	CD56	CD56	CD56
		BV510	Zombie Acqua	Zombie Acqua	Zombie Acqua

### Metabolic analysis in NK cells by respirometry (Seahorse)

We isolated NK cells from whole blood in both groups using the EasySep™ Direct Human NK cell isolation kit (STEMCELL Technologies, Vancouver, CA). We then plated these cells in a 6-well miniplate from Agilent (Santa Clara, CA), with a density of 150,000 cells. This density was chosen as it was found to have the best response for the experiments after testing. To measure the oxygen consumption rate (OCR) in mitochondria, we used the MitoStress assay with the drugs oligomycin, carbonyl cyanide m-chlorophenylhydrazone (FCCP), rotenone, and antimycin.

Briefly, oligomycin was the first injection used after taking basal measurements. This injection inhibits ATP synthase or complex V, thereby decreasing electron flow through the electron transport chain (ETP). This reduction in mitochondrial respiration is linked to cellular ATP-linked respiration. The next injection was FCCP (carbonyl cyanide-p-trifluoromethoxyphenylhydrazone), an uncoupling agent that disrupts the mitochondrial membrane potential and collapses the proton gradient. This injection allows electron flow through the ETC to be uninhibited, leading to maximum oxygen consumption by complex IV. The OCR stimulated by FCCP can then be used to calculate spare respiratory capacity, which is the difference between maximal and basal respiration. This measure indicates the cells ability to respond to increased energy demands or stress. The third injection was a mixture of a complex I (rotenone) and III (antimycin A) inhibitor. This combination shuts down mitochondrial respiration and enables the calculation of nonmitochondrial respiration driven by processes outside the mitochondria. The drug concentrations were standardized to ensure optimal trial performance. All analyses were conducted using the XF HS Mini Seahorse Analyzer (Santa Clara, CA) in collaboration with the Excellence Cluster Cardio-Pulmonary Institute (CPI) at Justus-Liebig University in Giessen, Germany.

### Statistical analysis

Data distribution was assessed using the Shapiro-Wilk test. For variables with normal distribution, parametric tests were applied; for non-normally distributed variables, non-parametric tests were used. Comparisons between untrained and endurance-trained groups were performed using either an unpaired t-test (for normally distributed data) or the Mann-Whitney U test (for non-normally distributed data), as appropriate. Specifically, the comparison of NK cells phenotypes after isolation versus 14 days of expansion (Fig. 1G) and Seahorse metabolic data (Fig. 6) were analyzed using these tests.

To assess the effects of group (untrained vs. endurance-trained), propranolol or rapamycin treatments (propranolol alone and PMA + propranolol; rapamycin alone and PMA + rapamycin), and their interaction (group × propranolol; group × rapamycin) a two-way repeated-measures ANOVA was performed for NK cell phenotypic outcomes. For dose-response experiments, two-way repeated-measures ANOVA was used to evaluate the effects of drug concentration (propranolol or rapamycin), stimulation (PMA), and their interaction (propranolol × stimulus; rapamycin × stimulus) on NK cell phenotypes. When a significant interaction effect was detected, Bonferroni-adjusted post hoc tests were conducted for multiple comparisons. If no significant interaction was observed, post hoc analyses were not performed.

Detailed *P*-values for interaction and main effects are reported in Supplementary Table 1. Statistical significance was defined as *P* < 0.05. All analyses were conducted using IBM SPSS (version 21, IBM Corp.), and graphical representations were created with GraphPad Prism (version 10).

## Electronic supplementary material

Below is the link to the electronic supplementary material.

Supplementary Material 1

## Data Availability

The data supporting the findings of this study are available from the corresponding author upon reasonable request.
