# Supplementary material for: Natural killer cells from endurance-trained older adults show improved functional and metabolic responses to adrenergic blockade and mTOR inhibition
Source: Sci Rep. 2025 Jul 14;15:25380. doi: 10.1038/s41598-025-06057-y (PMC12259902; doi:10.1038/s41598-025-06057-y)
Supplement: Supplementary file 1 — Supplementary Material 1 [file 41598_2025_6057_MOESM1_ESM.docx]

**Supplementary files**

**Supplementary Table 1.** Two-way repeated-measures ANOVA results for dose-response experiments evaluating the effects of propranolol, the stimulus (PMA), and their interaction (propranolol × stimulus) on NK cell phenotypes.

| Variables | Propranolol × stimulus interaction | | Propranolol | | Stimulus | |
| --- | --- | --- | --- | --- | --- | --- |
|  | *F* | *P-value* | *F* | *P-value* | *F* | *P-value* |
| CD3⁻CD56^+^ | 2.06 | 0.114 | 21.93 | 0.0006 | 35.18 | 0.004 |
| cytotoxic (CD56^dim^CD16^bright^) | 14.76 | P<0.0001 | 60.72 | P<0.0001 | 10.17 | 0.033 |
| effector (CD56^bright^CD16^dim^) | 14.92 | P<0.0001 | 63.44 | P<0.0001 | 11.45 | 0.028 |
| CD56^+^CD38^+^ | 112.7 | P<0.0001 | 294.5 | 0.0006 | 0.08 | 0.798 |
| CD56^+^CD57^+^ | 34.76 | P<0.0001 | 25.26 | 0.001 | 22.19 | 0.009 |
| CD56^+^CD107a^+^ | 9.93 | P<0.0001 | 37.96 | P<0.0001 | 6.20 | 0.067 |
| CD56^+^KLRG1^+^ | 4.40 | 0.022 | 15.11 | 0.0002 | 32.43 | 0.029 |
| CD56^+^LAG-3^+^ | 23.37 | P<0.0001 | 75.72 | 0.0004 | 30.58 | 0.005 |
| CD56^+^NKG2A^+^ | 0.92 | 0.488 | 6.60 | 0.0009 | 0.02 | 0.899 |
| CD56^+^NKG2D^+^ | 45.02 | P<0.0001 | 2.05 | 0.156 | 4.07 | 0.181 |
| CD56^+^PD-1+ | 7.92 | 0.0003 | 1.88 | 0.143 | 14.22 | 0.020 |
| CD56^+^TIM-3+ | 352.2 | P<0.0001 | 548.9 | P<0.0001 | 0.72 | 0.485 |

**Supplementary Table 2.** Two-way repeated-measures ANOVA results evaluating the effects of group, stimulus (propranolol/PMA), and their interaction (group × stimulus) on NK cell phenotypes in trained versus untrained older groups.

| Variables | Group × Stimulus interaction | | Stimulus | | Group | |
| --- | --- | --- | --- | --- | --- | --- |
|  | *F* | *P-value* | *F* | *P-value* | *F* | *P-value* |
| CD3⁻CD56^+^ | 3.79 | 0.003 | 4.77 | 0.0005 | 2.96 | 0.098 |
| cytotoxic (CD56^dim^CD16^bright^) | 3.00 | 0.014 | 3.22 | 0.026 | 5.08 | 0.033 |
| effector (CD56^bright^CD16^dim^) | 3.22 | 0.009 | 3.35 | 0.022 | 4.59 | 0.042 |
| CD56^+^CD38^+^ | 1.08 | 0.389 | 0.91 | 0.489 | 0.26 | 0.631 |
| CD56^+^CD57^+^ | 4.18 | 0.001 | 2.00 | 0.083 | 4.53 | 0.044 |
| CD56^+^CD107a^+^ | 3.85 | 0.003 | 4.06 | 0.002 | 7.34 | 0.012 |
| CD56^+^KLRG1^+^ | 8.01 | P<0.0001 | 1.83 | 0.113 | 0.027 | 0.871 |
| CD56^+^LAG-3^+^ | 5.31 | 0.002 | 2.77 | 0.040 | 15.40 | 0.011 |
| CD56^+^NKG2A^+^ | 0.43 | 0.827 | 3.23 | 0.009 | 2.26 | 0.146 |
| CD56^+^NKG2D^+^ | 3.31 | 0.008 | 3.44 | 0.006 | 3.34 | 0.080 |
| CD56^+^PD-1+ | 5.86 | P<0.0001 | 1.07 | 0.379 | 4.47 | 0.045 |
| CD56^+^TIM-3+ | 6.73 | 0.0003 | 12.29 | P<0.0001 | 0.32 | 0.595 |

**Supplementary Table 3.** Two-way repeated-measures ANOVA results for dose-response experiments evaluating the effects of rapamycin, the stimulus (PMA), and their interaction (rapamycin × stimulus) on NK cell phenotypes.

| Variables | Rapamycin × Stimulus interaction | | Rapamycin | | Stimulus | |
| --- | --- | --- | --- | --- | --- | --- |
|  | *F* | *P-value* | *F* | *P-value* | *F* | *P-value* |
| CD3⁻CD56^+^ | 12.43 | P<0.0001 | 7.47 | 0.0005 | 0.424 | 0.539 |
| cytotoxic (CD56^dim^CD16^bright^) | 8.97 | 0.0001 | 8.09 | 0.0003 | 0.395 | 0.553 |
| effector (CD56^bright^CD16^dim^) | 12.81 | P<0.0001 | 11.36 | P<0.0001 | 0.894 | 0.381 |
| CD56^+^CD38^+^ | 42.91 | P<0.0001 | 54.36 | P<0.0001 | 1.42 | 0.356 |
| CD56^+^CD57^+^ | 12.53 | P<0.0001 | 31.22 | P<0.0001 | 12.07 | 0.025 |
| CD56^+^CD107a^+^ | 3.87 | 0.022 | 1.21 | 0.343 | 1.29 | 0.319 |
| CD56^+^KLRG1^+^ | 10.13 | 0.0003 | 7.47 | 0.001 | 0.07 | 0.800 |
| CD56^+^LAG-3^+^ | 23.74 | 0.0002 | 3.93 | 0.047 | 1.87 | 0.304 |
| CD56^+^NKG2A^+^ | 12.25 | P<0.0001 | 17.96 | P<0.0001 | 1.41 | 0.301 |
| CD56^+^NKG2D^+^ | 3.92 | 0.021 | 4.77 | 0.010 | 2.52 | 0.187 |
| CD56^+^PD-1+ | 4.686 | 0.012 | 10.28 | 0.0003 | 33.91 | 0.004 |
| CD56^+^TIM-3+ | 3.21 | 0.075 | 20.24 | 0.0003 | 0.07 | 0.812 |

**Supplementary Table 4.** Two-way repeated-measures ANOVA results evaluating the effects of group, stimulus (rapamycin/PMA), and their interaction (group × stimulus) on NK cell phenotypes in trained versus untrained older groups.

| Variables | Group × Stimulus interaction | | Rapamycin | | Group | |
| --- | --- | --- | --- | --- | --- | --- |
|  | F | P-value | F | P-value | F | P-value |
| CD3⁻CD56+ | 9.25 | P<0.0001 | 23.53 | P<0.0001 | 0.0001 | 0.998 |
| cytotoxic (CD56^dim^CD16^bright^) | 4.03 | 0.003 | 4.40 | 0.002 | 4.40 | 0.053 |
| effector (CD56^bright^CD16^dim^) | 3.39 | 0.008 | 2.92 | 0.019 | 4.78 | 0.046 |
| CD56^+^CD38^+^ | 0.21 | 0.956 | 0.32 | 0.895 | 0.0001 | 0.990 |
| CD56^+^CD57^+^ | 10.04 | P<0.0001 | 38.64 | P<0.0001 | 0.85 | 0.386 |
| CD56^+^CD107a^+^ | 11.04 | P<0.0001 | 20.43 | P<0.0001 | 5.62 | 0.049 |
| CD56^+^KLRG1^+^ | 4.24 | 0.008 | 3.36 | 0.023 | 0.15 | 0.719 |
| CD56^+^LAG-3^+^ | 16.97 | P<0.0001 | 16.45 | P<0.0001 | 4.08 | 0.114 |
| CD56^+^NKG2A^+^ | 97.83 | P<0.0001 | 204.09 | P<0.0001 | 8.96 | 0.024 |
| CD56^+^NKG2D^+^ | 4.85 | 0.002 | 14.36 | P<0.0001 | 1.06 | 0.337 |
| CD56^+^PD-1+ | 4.10 | 0.010 | 10.15 | P<0.0001 | 0.33 | 0.593 |
| CD56^+^TIM-3+ | 3.10 | 0.031 | 3.26 | 0.026 | 3.21 | 0.148 |
